# Cefotaxime Resistant *Escherichia coli* Collected from a Healthy Volunteer; Characterisation and the Effect of Plasmid Loss

**DOI:** 10.1371/journal.pone.0084142

**Published:** 2013-12-27

**Authors:** Miranda Kirchner, Manal AbuOun, Muriel Mafura, Mary Bagnall, Theresa Hunt, Christopher Thomas, Jan Weile, Muna F. Anjum

**Affiliations:** 1 Department of Bacteriology and Food Safety, Animal Health and Veterinary Laboratories Agency, Addlestone, Surrey, United Kingdom; 2 School of Biosciences, University of Birmingham, Edgbaston, Birmingham, United Kingdom; 3 Institute for Laboratory and Transfusion Medicine at the Heart and Diabetes Centre, University Hospital of the Ruhr University, Bochum, Germany; University of Padova, Medical School, Italy

## Abstract

In this study 6 CTX-M positive *E. coli* isolates collected during a clinical study examining the effect of antibiotic use in a human trial were analysed. The aim of the study was to analyse these isolates and assess the effect of full or partial loss of plasmid genes on bacterial fitness and pathogenicity. A DNA array was utilised to assess resistance and virulence gene carriage. Plasmids were characterised by PCR-based replicon typing and addiction system multiplex PCR. A phenotypic array and insect virulence model were utilised to assess the effect of plasmid-loss in *E. coli* of a large multi-resistance plasmid. All six *E. coli* carrying *bla*_CTX-M-14_ were detected from a single participant and were identical by pulse field gel electrophoresis and MLST. Plasmid profiling and arrays indicated absence of a large multi-drug resistance (MDR) F-replicon plasmid carrying *bla*TEM, *aadA4, strA*, *strB*, *dfrA17/19*, *sul1*, and *tetB* from one isolate. Although this isolate partially retained the plasmid it showed altered fitness characteristics e.g. inability to respire in presence of antiseptics, similar to a plasmid-cured strain. However, unlike the plasmid-cured or plasmid harbouring strains, the survival rate for *Galleria mellonella* infected by the former strain was approximately 5-times lower, indicating other possible changes accompanying partial plasmid loss. In conclusion, our results demonstrated that an apparently healthy individual can harbour *bla*_CTX-M-14_ *E. coli* strains. In one such strain, isolated from the same individual, partial absence of a large MDR plasmid resulted in altered fitness and virulence characteristics, which may have implications in the ability of this strain to infect and any subsequent treatment.

## Introduction

Cefotaximes are 3^rd^ generation cephalosporins that are commonly used for treatment of enteric infections [1]. However, cefotaximases encoded by the *bla*_CTX-M_ genes are a growing family of resistance genes, which are often but not exclusively associated with mobile elements in *Escherichia coli*. Currently 137 variants of CTX-M genes have been described (Lahey clinic, http://www.lahey.org/Studies/other.asp#table1, accessed on 06/02/13), which can be divided into groups based on their DNA sequence. The cefotaximases are characterised by their often preferential hydrolysis of the third generation cephalosporin, cefotaxime.

In the UK, the most prevalent CTX-M type in humans is *bla*_CTX-M-15_, which is present on a plasmid and associated with an *Escherichia coli* of multi-locus sequence type (MLST) ST131 [2]. Since its first detection the CTX-M-15 bearing *E. coli* ST131 has been found world-wide and is described as an epidemic strain. The CTX-M-15 gene is commonly associated with IncF type plasmids which are often multi-resistant plasmids, carrying resistance genes for many other antibiotics [3]. In the UK, these strains are a particular problem in the elderly and have been associated with urinary tract infections. The group 9 gene *bla*_CTX-M-14_ is commonly detected in Spain, and its spread is associated with the dissemination of an IncK plasmid carrying *bla*_CTX-M-14_ [4]. However, group 9 CTX-M enzymes constitute only 4% of the ESBL positive *E. coli* recovered from human clinical cases in the UK [5]. In the UK, *bla*_CTX-M-14_ has been detected in cattle and has also been associated with an IncK plasmid [6].

Many of the beta-lactamase genes have been traced to the chromosome of environmental bacteria, which are considered the probable source. The *bla*_CTX-M_ genes originated from strains of *Kluyvera* and have been mobilised and spread by plasmids, often in combination with other resistance genes. It is often considered that the acquisition of resistance through mutation or gaining of resistance elements may exert a fitness cost upon the host. There are many studies describing the detrimental effect of antimicrobial resistance upon fitness, demonstrated by a reduction in growth rate or reduced virulence in animal models [7]. However, the proliferation of antimicrobial resistance and the spread of these large plasmids, suggests that bacteria can overcome any detrimental effect that may be exerted. Some bacterial species have developed mechanisms to overcome the fitness cost of antibiotic resistance with compensatory mutations. For example, mutation of Initiation factor 2 in *Salmonella* Typhimurium overcomes the fitness cost associated with formyl methionine transferase (*fmt*) mutants, which confer resistance to the peptide deformylase inhibitor antibiotic class [8]. In *E. coli* the fitness cost associated with gyrase mutations resulting in fluorquinolone resistance can be diminished with additional mutations [9].

During a yearlong study examining the effect of amoxicillin and minocycline use on the human gut flora, *E. coli* and other Gram-negative aerobes were examined for resistance gene carriage using a DNA array (EU FP7 project ANTIRESDEV; www.ucl.ac.uk/antiresdev). Whilst examining these isolates several CTX-M-14 positive *E. coli* were recovered from an apparently healthy individual from a single time point from the placebo group. Therefore the aim of this study was to determine what other resistances were present in these strains; if they were present on mobile elements such as plasmids; and what effect the plasmids may be having on the virulence and fitness of these strains.

## Materials and Methods

### Bacterial isolation, culturing, and antibiotic sensitivity testing

During a study to investigate the effect of antibiotic (amoxicillin and minocycline) use on the gut flora, *E. coli* were isolated which were analysed for resistance gene content. Those positive for a *bla*_CTX-M_ gene were analysed further in this study. They were isolated from a faecal sample collected from a participant in the placebo group, and the sample was taken 12 months after the start of the study (EU FP7 project ANTIRESDEV; www.ucl.ac.uk/antiresdev). Ethical permission was obtained for all isolates collected for this study from participants from the South West London Research Ethics Committee. Written consent was obtained from participants to participate in this study, which was approved by the ethics committee. The reference for the ethical clearance for this study is 10/h0806/12. All isolates were recovered from agar containing either 0.5 mg/L minocycline (n = 2), 4 mg/L minocycline (n = 2), or 8 mg/L amoxicillin (n = 2). MALDI was performed to determine the identity of each isolate. Isolates were cultured on Luria-Bertani (LB) agar at 37°C for all applications except for the preparation of genomic DNA for array analysis where blood agar was utilised. *E. coli* were tested for resistance to a panel of 22 antimicrobial agents or combinations using the VITEK system (bioMérieux, France) and results interpreted according to Deutsches Insitut fur Normung E.V. (DIN) 58940 (http://global.ihs.com/standards.cfm). The panel consisted of; Amoxicillin (AMX), Amikacin (AMK), Amoxicillin/Clavulanate (AMC), Ampicillin (AMP), Cefalothin (CEF), Cefotaxime (CTX), Cefoxitin (FOX), Ceftazidime (CAZ), Cefpodoxime (CPD), Cefuroxime (CXM), Cefuroxime Axetil (CXM-AXETIL), Ciprofloxacin (CIP), Gentamicin (GEN), Imipenem (IPM), Meropenem (MEM), Nitrofurantoin (NIT), Norfloxacin (NOR), Piperacillin/Tazobactam (TZP), Piperacillin/Sulbactam (PIP-SUL), Tetracycline (TET), Tobramycin (TOB), and, Trimethoprim/Sulfamethoxazole (SXT). Resistance to Streptomycin (S; S10), compound sulphonamide (SUL; S3 300), Nalidixic acid (NA; NA30), Chloramphenicol (C; C30), and Gentamicin (GN; CN10) was determined according to previously described method [10].

### Antibiotic resistance and virulence gene array

A DNA array containing probes for >90 antibiotic resistance genes and approximately 50 validated virulence genes was utilised as described previously [11]–[13]. DNA was extracted by re-suspending a loopful of fresh bacterial growth in 400 µl of lysis buffer (containing 0.24 mg/l proteinase K, 0.05% Tween 20, 0.1 M Trizma-HCl), mixed and incubated in a Thermomixer for 2 h–4 h at 60°C with agitation at 550 rpm, followed by 95°C for 15 min. Cell debris was removed following centrifugation and supernatant used in subsequent steps. DNA was labelled as described previously using primers described in [11], [13]. Labelled DNA was hybridised to the array using the buffers provided in the HybPlus Kit (Alere Technologies, Jena, Germany) and for all steps, including hybridisation and washing steps the arrays were agitated at 550 rpm in a Thermomixer. Ten microlitres of labelled DNA was mixed with 90 µl C1 (hybridisation buffer), each array was prepared by washing in water, followed by C1 for 5 min at 65°C. Once the sample was added it was incubated for 1 h at 55°C, the array was than rinsed with C2 (wash buffer) and washed twice at 30°C for 5 min. Conjugate solution (C3) containing horse-radish peroxidase conjugated streptavidin was added for 15 min at 30°C, followed by three washes in wash buffer 2 (C5) by repeated pipetting. The presence of bound DNA was determined using Seramun Green (Buffer D1). After 10 min incubation at room temperature signals were read on an ArrayMate (Alere Technologies, Germany) using IconoClust software (Standard version; Alere Technologies, Germany). Mean signal intensities of two replicate spots per probe were used for analysis and values ≥0.5 were considered positive.

### Characterisation of the CTX-M positive *E. coli*

The CTX-M gene from an isolate representative of the CTX-M group 9 positive isolates was sequenced as described previously [14]. Pulsed-field gel electrophoresis was performed according to the method of PulseNet for *Escherichia coli* [15]. Genomic DNA was digested with *Xba*I restriction endonuclease prior to separation. Gel images were analysis using Bionumerics (Applied Math, Belgium) and clustering performed using the DICE co-efficient and UPGMA (Unweighted Pair Group Method with Arithmetic Mean).

Multi-locus sequencing typing (MLST) was performed using the loci, *adk*, *fumC*, *gyrB*, *icd*, *mdh*, *purA*, and, *recA*, according to the method of Wirth *et al*. [16]. Sequences were compared to the *E. coli* MLST database held at the University of Cork, Dublin (http://mlst.ucc.ie/mlst/dbs/Ecoli).

### Plasmid sizing, Incompatibility testing, and addiction system determination

Plasmid DNA was extracted from the isolates using the method of Kado and Liu [17]. DNA was separated on 0.8% agarose gels and visualised after staining with ethidium bromide, using a UV-transluminator. The approximate size of each plasmid was determined after comparison with an *E. coli* 39R861 [18], containing 4 plasmids of known size and a supercoiled DNA ladder (Sigma). Size determination was performed using Bionumerics software (Applied Math).

PCR–based plasmid replicon typing (PBRT) was performed as described [19] using a multiplex PCR kit from Diatheva (Fano, Italy) according to the manufacturer's instructions. The F replicon sequence was determined using the F_repB_-FW and F_repB_-RV described by Carattoli *et al* 2005 [19]. PCR was also used to determine the presence of the toxin-anti-toxin addiction systems in these isolates [20]. The method used is able to detect seven addiction systems (*pemk*, *hok-sok*, *ccdAB*, *relBE*, *vagCD*, *pndAC*, and *srnBC*). Primers and method were as described in Mnif *et al* 2010.

The F replicon from ARD1257 and ARD1258 were sequenced using the FrepB primers of Carattoli *et al* 2005 [19] using the same conditions described in the article.

### Transferability determination and plasmid curing

The ability of the *bla*CTX-M containing plasmid to be transferred was determined by in-broth conjugation studies using *Salmonella* Typhimurium 26R775 as a recipient and selecting trans-conjugants on Chromagar ECC containing 100 mg/l rifampicin and 1 mg/l cefotaxime [21]. Where conjugal-transfer was unsuccessful, the resistance plasmids were transferred to *E. coli* (DH10B, Invitrogen) by transformation [10]. Complementation of ARD1258C and ARD1257 with pARD1258 was attempted by electroporation, heat-shock and conjugation. Competent cells of ARD1258C and ARD1257 were prepared using either ice-cold water/glycerol for electroporation or rubidium chloride for heat-shock. Conjugal-transfer of pARD1258 was also attempted as described above [22].

ARD1258 was cured of a plasmid using the pCURE plasmid displacement system according to a published method [23]. The plasmid used to displace the F-replicon plasmid in this study was pCURE™2 [23] as described in the PlasGene pCURE™2 kit (Plasgene Ltd, Birmingham, UK) generating ARD1258C.

### Growth characteristics

The growth of ARD1257, ARD1258, ARD1258C and MG1655 was assessed using the FLUOstar (BMG Labtech) in two different media. Overnight cultures of each isolate growth in LB-G at 37°C were diluted to 0.1% solution in LB-G or minimal media (1X MOPS Mixture, 1.32 mM K_2_HPO_4_and 0.4% Glucose) and 200 µl added to triplicate wells of a 96 well plate. The absorbance (600 nm) was measured for 24 h at 12 min intervals during incubation at 37°C. This was repeated on three occasions.

### Phenotypic microarray; Biolog analysis

The strains, ARD1257, ARD1258, ARD1258C and DH10B-pARD1258 were analysed using the Biolog phenotype microarray (PM) system (Hayward, CA), which enables a high throughput screen of bacterial response to 240 metabolic effector compounds [24]. Each isolate was analysed using 10 PM plates containing antimicrobials (PM11–14) and other metabolic effectors (PM15–20) in duplicate. All media, reagents, and PM panels were used according to manufacturer's instructions. Bacterial isolates to be tested were cultured for 16 h on LB agar plates at 37°C in aerobic conditions. Cells were re-suspended in 10 ml of inoculating fluid (IF-0a) and the optimal density adjusted to 85% transmittance. For each plate 12 ml of media IF-10a was mixed with 120 ul of bacterial cell suspension, which was also supplemented with a final concentration of 0.02 M sodium succinate/0.002 mM ferric citrate. Tetrazolium dye A supplied by Biolog Inc (Hayward, USA) was used to measure the cellular respiration. All microplates were incubated aerobically in the Omnilog instrument at 37°C and monitored for colour change at 15 min intervals for 120 hours. Kinetic data was analysed with OmniLog-PM software (Biolog). All experiments were performed in duplicate on different days and comparisons between strains were based on the average of the area under the curve values at 24 hours.

### Galleria mellonella virulence model

*Galleria mellonella* was used as a model to compare the *in vivo* virulence of ARD1257, ARD1258, ARD1258C with the laboratory *E. coli* MG1655 strain to determine if plasmid loss affected the ability of each strain to kill *Galleria mellonella* [25], with respect to a laboratory control strain. All bacterial strains including MG1655, a K-12 *E. coli* [26], were grown for approximately 18 h in LB broth with shaking at 37°C. Cells were harvested to give a final concentration of 10^8^ bacteria in 1 ml Phosphate-buffered-saline (PBS). This was then further diluted to 10^6^ and 10^4^ CFU/ml to provide a dose of 10^4^ CFU in 10 µl and 10^2^ CFU in 10 µl, respectively. Ten larvae were injected with a 10 µl dose of either 10^2^ or 10^4^ CFU in the right fore proleg using a Hamilton® syringe. All larvae were incubated at 37°C for 24 h before determining rates of survival. During each experiment 10 larvae were selected and were used as an uninfected control and 10 larvae were injected with sterile PBS alone. Each experiment was performed in triplicate. The exact dose was determined after plating the inoculum on LB agar. After 24 h incubation the survival rate in each group was determined and macroscopic appearance noted. In addition, the levels of *E. coli* in the larvae following 24 h were determined. Three larvae from each group during one experiment were individually homogenised in 1 ml PBS and a 10-fold dilution series prepared to determine the bacterial levels. Each group was plated on Chromagar ECC containing 2 mg/l cefotaxime with the exception of MG1655, which were plated on Chromagar ECC without antibiotics.

## Results

### Phenotypic and genotypic analysis of *bla*_CTX-M-14_ positive *E. coli*

A DNA array was employed to identify resistance genes in isolates collected during a study investigating the effect of antibiotic use on the gut flora in healthy volunteers. Six *E. coli* isolates were detected (from the same participant) which were positive for *bla*_CTX-M_ group 9 positive probe by microarray. One isolate, ARD1257, was only positive for *bla*_CTX-M_, while five of the isolates including ARD1258, were also positive for *tetB*, *strA*, *strB*, *bla*TEM, *aadA4*, *sul1*, and *dfrA17/19* by microarray. ARD1257 and ARD1258 were resistant to 12 beta-lactam antibiotics or beta-lactam/inhibitor combinations (Table 1). In addition, both were resistant to ciprofloxacin, norfloxacin and nalidixic acid. ARD1258 was also resistant to trimethoprim/sulphamethoxazole and tetracycline. The gene encoding CTX-M from strain ARD1257 was sequenced and it was confirmed as *bla*_CTX-M-14_ (NCBI reference: AF252622). PFGE analysis of all CTX-M-14 positive *E. coli* demonstrated they were indistinguishable by *XbaI*-PFGE (data not shown). ARD1257 and ARD1258 had identical MLST types both belonging to ST648. Virulence gene carriage was assessed using a DNA array containing *E. coli* virulence factors, and they were rare in these isolates. The *iss* gene, encoding increased serum resistance, was the only virulence gene detected by the array in these isolates and was present in all isolates tested.

**Table 1:** Characteristics of *bla*CTX-M positive *E. coli* collected from a participant.

	ARD1257	ARD1258	ARD1258C
**MLST type**	ST648	ST648	ST648
**Resistance genes**	*bla*CTX-M14	*bla*CTX-M14, *bla*TEM, *aadA4, strA*, *strB*, *dfrA17/19*, *sul1, tetB*	*bla*CTX-M14
**Phenotype**	AMX, AMC, AMP, CEF, CTX, CAZ, FOX, CPD, CXM, CXM-AXETIL, TZP, PIP/SUL, CIP, NOR	AMX, AMC, AMP, CEF, CTX, CAZ, FOX, CPD, CXM, CXM-AXETIL, TZP, PIP/SUL, CIP, NOR, SXT, S, SUL, TET, NA	AMP, AMC, CTX, NA, CIP*
**Replicon types**	IncFIB,FIA,FII	IncFIB,FIA,FII	-
**Addiction systems**	*pem*K, *ccd*AB, *hoksok*, *srn*BC	*pem*K, *ccd*AB, *hoksok*, *srn*BC	-
**Transferability**	Non-conjugative	Non-conjugative	N/A

*Note that ARD1258C was not tested using the Vitek system for the expanded panel of beta-lactams antibiotics, as performed for ARD1257 and ARD1258.

### Plasmid characteristics

**Figure 1:**
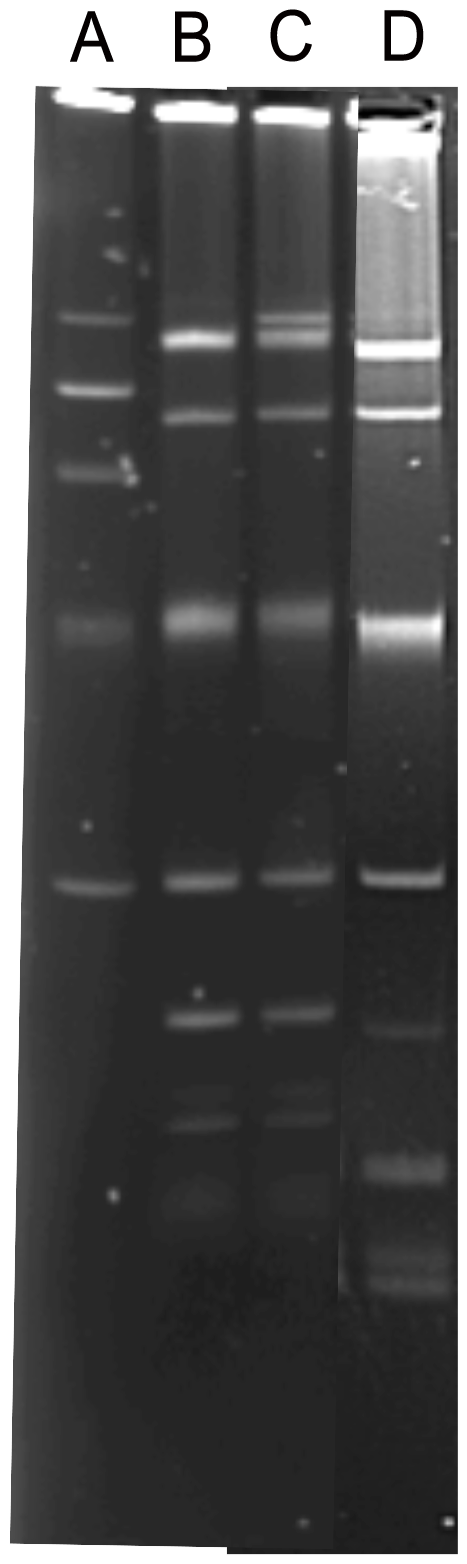
Gel picture showing the electrophoretic separation of plasmids. The plasmids carried by ARD1257 (B), ARD1258 (C), and ARD1258C (D) migrating in 0.8% agarose, for 270 min at 150 v, 4°C, are shown. Lane A shows the reference plasmid bands from strain 39R861. The reference plasmid bands are from top to bottom; 148 kb, 63 kb, 36 kb, genomic DNA band, 6.8 kb. Lane A–C were run on a separate occasion to lane D but under identical run conditions with a reference plasmid.

ARD1258 was selected for further analysis as a representative of the five isolates with identical resistance genes and PFGE profiles. ARD1257 was selected for further analysis due to the differences in its resistance gene carriage. Electrophoretic separation of the plasmids for each isolate showed presence of 6 bands for ARD1257 and 7 for ARD1258, which may be indicative of multiple plasmids. However, given the fact that plasmids may be present in more than one form each band may not represent a different plasmid. Nevertheless, ARD1257 lacked a large band of approximately 140 kilo bases (kb) compared to ARD1258 (Figure 1) and the other isolates (not shown). Only three F replicon types were detected by PCR (see Table 1) in both ARD1257 and ARD1258. This suggested that some of the plasmids were of incompatibility types not detectable by the Carattoli et al PCR (27) and that ARD1257 and ARD1258 both contained multiple F-replicons. Sequence analysis of the F-replicon from ARD1257 and ARD1258 demonstrated that the F-rep sequences were identical. Both sequences were also identical to the F-replicon sequence of pRSB107 (GenBank accession: AJ851089). Both also carried the *pemK*, *ccdAB*, *hoksok*, and *srnBC* addiction systems, as determined by PCR.

A plasmid cured form of ARD1258 was generated using the pCURE2 system which can eliminate F-replicon plasmids carrying the addiction systems, *ccdAB* and *hok/sok*. Bacteria lacking pCURE2 were then selected based on sucrose resistance since pCURE carries *sacB* that confers sucrose sensitivity. ARD1258C was generated to compare its characteristics with the isolate ARD1257. Following curing, ARD1258C had lost a band of approximately 140 kb, identical in size to the band missing from ARD1257 but still retained the several other bands which were mostly similar to those in ARD1257 and ARD1258 (Figure 1.). ARD1258C was negative by PCR for all F replicons and addiction systems following curing, indicating presence of these genes on the large MDR plasmid (Table 1). ARD1258C was also analysed by array to determine the gene content post-curing. ARD1258C was positive for the *bla*_CTX-M_ group 9 probe but had lost all other resistance genes, being identical to ARD1257. ARD1258C also became sensitive to TET, SXT, SUL, and S following curing confirming the loss of these resistance genes. This led us to conclude that a large 140 kb multi-drug resistance (MDR) plasmid had been lost from ARD1258C, which was only partially lost from ARD1257 due to presence of the F-rep and addiction systems in the latter.

We attempted to complement ARD1257 and ARD1258C with the 140 kb MDR plasmid (named pARD1258) by electroporation, heat-shock and conjugation, but this was unsuccessful. However, we were able to transfer pARD1258 to DH10b competent cells (Invitrogen) by electroporation. Microarrays were performed and confirmed presence of *bla*TEM, *aadA4, strA*, *strB*, *dfrA17/19*, *sul1, tetB* resistance genes and plasmid profiling showed presence of a 140 kb plasmid (data not shown). PCR confirmed that the plasmid transferred to DH10b was Frep positive and carried *pemK*, *ccdAB*, *hoksok*, and *srnBC* addiction systems.

### Altered fitness characteristics and virulence; the effect of plasmid loss

**Figure 2:**
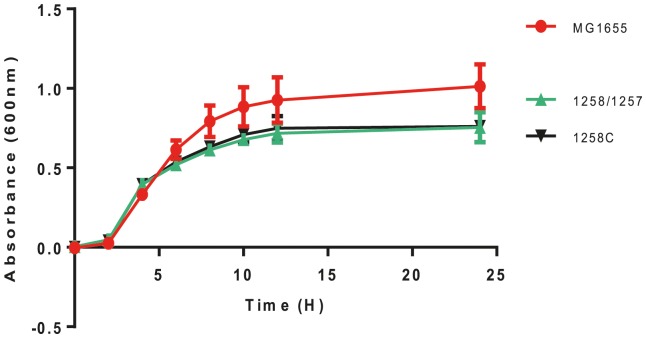
Growth curves in LB media. The growth curves for ARD1258, ARD1257, ARD1258C and MG1655 determined over 24°C in LB media are shown. The absorbance in corrected for the blank and represents the mean of triplicate wells from three individual experiments; error bars indicate a 95% confidence interval.

The growth of ARD1257, ARD1258, ARD1258C and MG1655 was compared by monitoring change in absorbance over a 24 h period when grown in either LB or MOPS minimal media. Rate of growth differed very little between all isolates, with ARD1257 and ARD1258 being identical and ARD1258C growing fractionally better in LB broth (Figure 2.) or minimal media (data not shown). All 3 test strains differed slightly from the control MG1655 and had a lower growth yield at 24 h in LB.

To investigate if there were any other effect of plasmid loss on the fitness of *E. coli* harbouring *bla*_CTX-M-14_ gene Omnilog phenotypic microarrays were utilised. The aim was to determine if ARD1257, which at least partially lacked the large MDR plasmid had differential respiration characteristics in the presence of different antibiotics and stress modulators, compared to ARD1258, or DH10b-pARD1258 (for a selected subset). Table 2 shows the area under the curve (AUC) values for ARD1257 and ARD1258 for compounds where a significant difference was observed. Both ARD1257 and ARD1258C (data not shown) demonstrated similar respiration profiles for these compounds. Each array was performed at least in duplicate and a T-test was used to compare the averages of each replicate AUC for each compound. This allowed the identification of compounds where a significant difference in AUC values between ARD1257 and ARD1258 were observed. An AUC value <14 is considered negative i.e. the bacterium is unable to respire in the presence of the compound, while values >14 were considered positive. Both ARD1257 and ARD1258C were unable to respire in the presence of the several tetracycline derivatives including chlortetracycline, demeclocyline, minocycline and rolitetracycline, with AUC values of 12 or less (Table 2.). This is in agreement with the genotype array results demonstrating the loss of *tetB* in ARD1257 and ARD1258C. Both strains were also unable to respire in the presence of trimethoprim and members of the sulphonamide antibiotic class (sulfadiazine and sulfisoxazole), due to the loss of *dfr17/19* and *sul1*, respectively. In addition, ARD1257 and ARD1258C were unable to respire in the presence of 2,4-Diamino-6,7-diisopropylpteridine (O129), chlorhexidine, and 8-hydroxyquinoline, which may not be directly related to changes in resistance gene carriage (Table 2). ARD1258 and ARD1257 were able to respire in the presence of streptomycin in up to 10 mg/l streptomycin (PM16 E01-E04) with mean AUC values >14, indicating that although *strA* and *strB* were lost from ARD1257, this strain was still able to respire at a concentration which is just above the British Society for Antimicrobial Chemotherapy (BSAC) breakpoint to streptomycin resistance in *E. coli* is 8 mg/l.

**Table 2:** Compounds identified using the Omnilog Phenotype Microarray (Biolog), where a significant difference in respiration, denoted by AUC values, were detected between ARD1257 and ARD1258.

Phenotype-function	Genotype	Compound (Plate and well location)	Mean area under the curve values (AUC)		P = values
			ARD1257	ARD1258	
Tetracycline	*tet*B	Chlortetracycline (PM11C A08)	11.48	14.51	0.00442
Minocycline		Minocycline (PM11C C11)	11.51	14.22	0.00071
Tetracycline	*tet*B	Demeclocycline (PM11C D08)	11.02	14.51	0.00003
Tetracycline	*tet*B	Penimepicycline (PM12B B08)	11.37	14.51	0.00379
Vibriostatic agent	N/A	2,4-Diamino-6,7-diisopropylpteridine (PM12B E04)	11.69	14.67	0.00406
Sulphonamide	*sul*1	Sulfadiazine (PM12B E08)	12.89	14.45	0.03618
Tetracycline	*tet*B	Doxycycline (PM13B C08)	11.38	14.58	0.00010
Tetracycline	*tet*B	Rolitetracycline (PM13B D12)	12.18	14.68	0.00002
Trimethoprim	*dfr*19	Trimethoprim (PM16A B12)	12.37	14.59	0.00548
Sulphonamide	*sul*1	Sulfisoxazole (PM18C C08)	12.88	14.48	0.00466
Antiseptic	N/A	Chlorhexidine (PM19 C04)	12.00	14.44	0.08951
Tetracycline	*tet*B	Oxytetracycline (PM20B F08)	11.84	14.56	0.00419
Antiseptic	N/A	8-Hydroxyquinoline (PM20B G11)	12.25	14.23	0.01804

T-Test performed to compare AUC values for ARD1257 and wild-type ARD1258.

The plasmid pARD1258 was introduced into DH10b and Biolog phenotype microarrays was performed for selected plates (PM11C, PM19 and PM20). It was found DH10B/pARD1258 was resistant to tetracycline compounds present in PM11C and PM20B, similar to ARD1258; while DH10B was sensitive to these like ARD1257 and ARD1258C. DH10b and DH10b/pARD1258 were more sensitive to chlorhexidine than ARD1258 and ARD1257; both were unable to respire in the highest concentration of chlorhexidine. Nevertheless, there was a difference in their ability to respire in the second highest concentration where presence of the plasmid allowed respiration of DH10b/pARD1258, which DH10b was unable to perform. However, a difference in mean AUC values were not seen between DH10b and DH10b/pARD1258 for 8-hydroxyquinoline, both were able to respire in its presence (data not shown).

**Figure 3:**
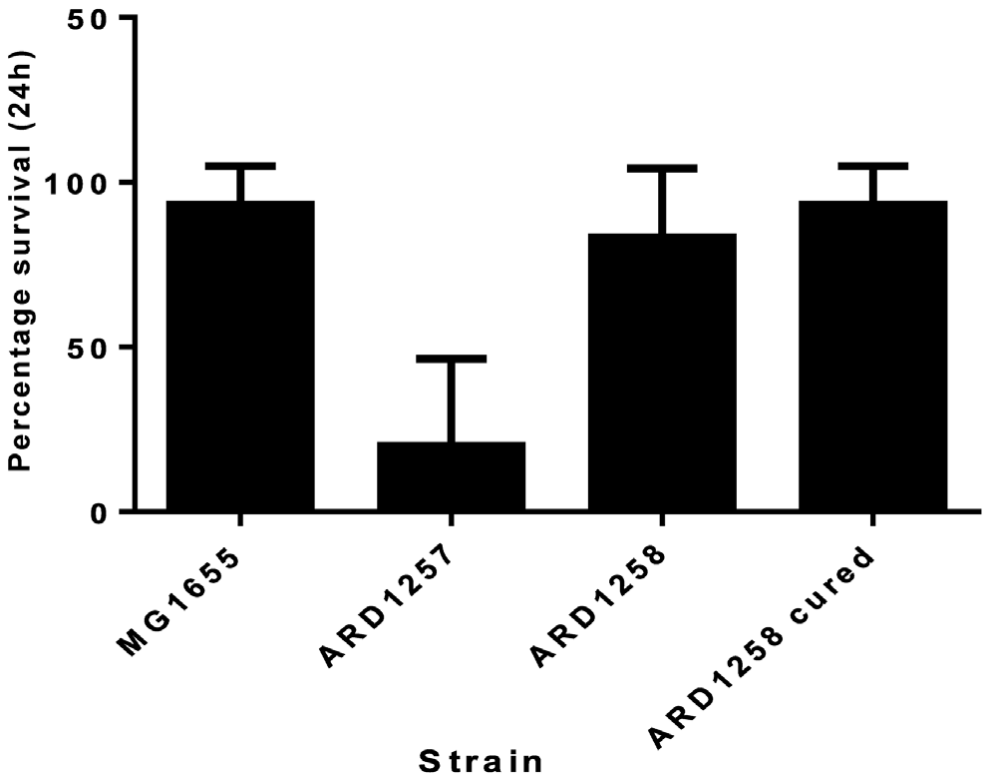
Survival of *Galleria mellonella* with *E. coli* isolates. The mean percentage survival rate of *Galleria mellonella* infected with different *E. coli* strains after 24 h. Error bars indicate the Standard deviation from replicate experiments.

**Figure 4:**
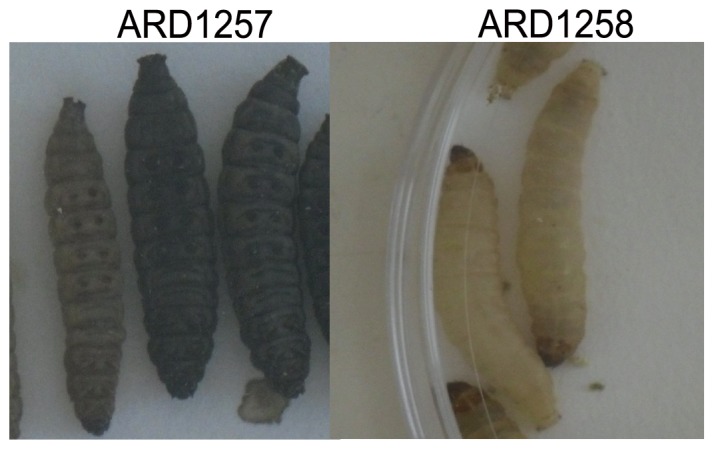
Difference in melanisation of *Galleria mellonella* following infection with *E. coli* isolates. Images of *Galleria mellonella* infected with ARD1257 and ARD1258, demonstrating the melanisation that occurred following infection with ARD1257.

To further assess the differences between ARD1258 and cured strains a virulence model was utilised. *Galleria mellonella* the larvae of the Greater Wax moth were infected with each strain to determine their ability to kill the larvae. This model has been used to assess the virulence of different bacterial species including enteropathogenic *E. coli* [27]. ARD1257, ARD1258, and ARD1258C were assessed in the model using 10^2^ CFU/larvae. The mean rate of survival of *Galleria* at this bacterial dose was strain dependant and infection with ARD1257 demonstrated the lowest rate of survival at an average of 20% (range 0–50%) (Figure 3). While for ARD1258 and ARD1258C the mean survival rates for *Galleria* were 83.3% (range 60–100%) and 93.3% (range 80–100%), respectively. MG1655 was used as a control in this study and had a survival rate of 93.3% (range 80–100%). Therefore, the mean rate of survival for ARD1257 infected larvae was up to 5 times lower compared to ARD1258 and ARD1258C, respectively. At a higher infection dose of 10^4^ CFU/larvae the survival rate of the larvae was lower for all strains, although larvae infected with ARD1257 was still lower than ARD1258 or ARD1258C (data not shown). A colour change was observed in some larvae following 24 h incubation, as previously described [25]. Those killed following infection were black or dark brown while surviving larvae were identical to the uninfected controls (Figure 4.). Diarrhoea was observed in larvae infected with ARD1257, ARD1258, and to a lesser extent in those infected with ARD1258C. A quantitative measure of diarrhoea was not possible. During one experiment an estimation of the level of internalised *E. coli* was made. Three larvae infected with ARD1257, ARD1258 and ARD1258C were homogenised and bacterial counts performed. The levels of bacteria (after 24 h incubation with a dose of 10^2^ CFU/larvae) in ARD1257 and ARD1258 were 3.9×10^7^ CFU/larvae and 2.85×10^3^ CFU/larvae, respectively. While for ARD1258C on average of 10^2^ CFU/larvae were detected. DH10b and DH10b/pARD1258 were also tested in this model and all *Galleria* survived with and without the plasmid, following infection at a dose of 10^2^.

## Discussion

During a study to assess the effect of amoxicillin or minocycline treatment on the gut flora, *E. coli* were recovered from a healthy volunteer carrying *bla*_CTX-M-14_. All 6 isolates were identical by PFGE and MLST and were isolated at month 12 of the study. As these *E. coli* were first detected at month 12 of the yearlong clinical study it was not possible to determine the ability of this strain to persist in the human gut, and its effect if any, on the host. We also detected *bla*_CTX-M-15_ positive *E. coli* during this study from a different participant which was able to persist for 4 months (data not shown). CTX-M positive *E. coli* were detected in 5% of the participants included in this study, although screening was not specifically designed to assess carriage of *bla*_CTX-M_ genes. A study from 2012 in the UK found that the community carriage of *bla*_CTX-M_ was 11.3%, slightly higher than seen in Europe (8.1%) [28].

The *E. coli* carrying *bla*_CTX-M-14_ isolates were further characterised and were shown to be multi-resistant (with the exception of the partially cured strain), carrying several plasmids encoding antibiotic resistance genes. It is worth noting that the electrophoretic separation method used to profile plasmids [17] in this study cannot distinguish between different forms of the same plasmid (e.g. linear, supercoiled, relaxed, concatenated) being present, so we cannot be entirely certain of the number of plasmids present. However, it is evident from comparing ARD1258 and ARD1258C that curing has resulted in the loss of a 140 kb band on the gel; there were several other bands present on the gel and both isolates were positive for *bla*_CTX-M-14_ indicating at least one other plasmid than the 140 kb MDR-plasmid to be present in these strains.

However, location of *bla*_CTX-M-14_ was not determined in this study as it was not possible to transfer a plasmid harbouring this gene either by conjugation or transformation into other *Salmonella* or *E. coli* strains (data not shown). Replicon typing detected only three F-replicons in isolates ARD1257 and ARD1258 and suggesting that *bla*_CTX-M-14_ is either carried on an IncF plasmid or a plasmid with a replicon type not detectable with the PCR-based method. In a previous study, the spread of *bla*_CTX-M-14_ bearing strain in Spain has been associated with IncK type plasmids [4], while a recent study of UK human *E. coli* carrying *bla*_CTX-M-14_ found that approximately 20% of the isolates had IncK type plasmids and the remaining isolates had plasmids of different incompatibility groups [5]. IncK was not detected in these isolates by PCR (data not shown) and therefore *bla*_CTX-M-14_ must be associated with a plasmid of a different Incompatibility group.

During this study a single *E. coli* was detected which lacked elements of a large plasmid carrying multiple antibiotic resistance determinants. Electrophoretic separation of plasmid DNA from ARD1257 and ARD1258 demonstrated that ARD1257 was lacking a large plasmid band of around 140 kb and it had lost the resistance elements encoded by this plasmid. However, the F replicons and addiction systems which were identical to that in ARD1258 remained in ARD1257. Following curing of the 140 kb plasmid in ARD1258C all F replicon and addictions systems were lost, indicating that they were all co-localised on the same 140 kb plasmid. This suggests that although the plasmid band has been lost from ARD1257 some elements of the plasmid which included the F-replicon and addiction systems remained. Southern blot analysis was performed on plasmid DNA that had been electrophoretically separated on a gel and probed with a 270 bp amplicon from the *repF* gene. The results confirmed presence of the F-replicon in both ARD1258 and ARD1257. Although there was a difference in the size of the band to which the probe hybridised in the two strains we were not clearly able to distinguish the location of the F-replicon in ARD1257 (data not shown).

As ARD1257 appears to have lost a large resistance gene element, we used this as an opportunity to assess the effect of resistance gene carriage on fitness. Therefore, we assessed the ability of ARD1257 strain to respire in different compounds and its virulence in an insect model. Phenotypic array analysis demonstrated that ARD1257 and the artificially cured ARD1258C strains were very similar in their phenotypic characteristics relating to compounds in which they could respire. Loss of ability to respire in the presence of sulphonamide, tetracycline, and trimethoprim type compounds corresponded to the loss of the 140 kb plasmid encoding *sul*1, *tet*B, and *dfr17/*19, respectively. Although *strA*-*str*B were associated with loss of the 140 kb plasmid in ARD1257, both ARD1257 and ARD1258C respired in the presence of up to 10 mg/l streptomycin, which is just above the BSAC breakpoint for Streptomycin. As streptomycin is a bactericidal agent this result suggested other resistance mechanisms were present but this was not investigated further during this study. Isolates missing the resistance element (ARD1257 and ARD1258C) were also unable to respire in the presence of the antiseptics chlorhexidine at the same concentration as ARD1258. Although isolate DH10B-pARD1258 was also unable to respire in chlorhexidine at the same concentrations as ARD1258 it was able to respire in higher levels of chlorhexidine than DH10B alone. Chlorhexidine is a commonly used biocide which can be found in many everyday products used in the home, resistance to this compound will enable the bacteria to survive and spread in this background. The mechanisms of biocide resistance can include efflux pumps, and for chlorhexidine an efflux pump, *cepA*, has been reported on the chromosome of *Klebsiella pneumonia* [29]. Morita *et al* (2003) demonstrated that chlorhexidine was able to induce the chromosomally located *mexCD*-*oprJ* efflux pump in *Pseudomonas aeruginosa* [30]. Plasmid-mediated mechanisms of chlorhexidine resistance have not been described in Gram-negative bacteria. The results of this study suggest that the ability to respire in the presence of this compound is associated with presence of the resistance elements found on the 140 kb plasmid and some other characteristics of the strain. Further studies are necessary to confirm if the presence of this plasmid is indeed enabling growth in chlorhexidine and the underlying mechanism. There was also a difference in levels of respiration for ARD1257 and ARD1258 in the presence of a second antiseptic, 8-Hydroxyquinoline. It was not possible to confirm if the plasmid alone was responsible for this phenotype as DH10b was also able to respire in the presence of this compound at the same levels. In addition isolates carrying the 140 kb plasmids were better able to respire in the presence of the vibriostatic agent (O/129). Resistance to O/129 has been linked to trimethoprim resistance in some bacterial species including *Pasteurella* spp. [31] and is due to cross-resistance by dihydrofolate reductase (DHFR), therefore the loss of *dfr17/*19 in strains partially or fully lacking the plasmid (i.e. ARD1257 and ARD1258C) may account for this difference.

Although both cured strains showed the same phenotype using the Omnilog phenotype microarrays, there were differences using the *Galleria mellonella* virulence model. Isolate ARD1257, was 5 times more virulent in this model than the parent strain or the isolate cured using the pCURE system. ARD1257 was able to grow *in vivo* to 10^4^ and 10^5^ times higher numbers than the other two strains which may indicate that ARD1257 is overloading the larvae with bacteria causing death, or this strain harbours additional virulence genes that were not detected by our array. The difference in *in vivo* growth was particularly interesting as no difference was seen in the *in vitro* growth rates of isolates with or without the plasmid, in rich or Glucose minimal media. A study by Leuko and Raivio (2012) [27] supports our conclusions; they also found that the levels of wild-type bacteria in the larvae increased as the percentage survival of larvae decreased. Larvae infected with ARD1257 and those killed in the other infected groups underwent melanisation, which is due to the activation of prophenoloxidase, which in turn causes melanin production. *Galleria mellonella* produce melanin is response to bacterial infections as a mechanism to kill the invading organism [32]. It is noteworthy that the plasmid alone did not confer advantage or disadvantage for virulence as evident from *Galleria mellonella* studies performed comparing DH10b and DH10b/pARD1258.

Therefore from our virulence model experiments the effects of resistance gene loss was less clear as a strain that had lost the resistance gene element but had retained part of the plasmid (including the F replicon and addition systems) showed enhanced virulence, which was not seen when an artificially cured strain was included (ARD1258C) or a strain only harbouring the plasmid (DH10b/pARD1258) but in a different background. It indicated that ARD1257 had other genetic changes in addition to those that could be attributed to loss of resistance genes. It may be speculated that the remnant plasmid has integrated somewhere into the bacterial genome that results in derepression or inactivation of genes consequently increasing virulence of ARD1257. Although these genomic changes are not reflected by the PFGE profile it warrants further exploration by whole genome sequencing to help explain the change in virulence and the ability of ARD1257 to grow to higher levels in the *Galleria*.

It is well known that in nature mobile genetic elements such as plasmids are easily acquired and lost from bacterial cells [33]. Therefore, the partial or full loss of a MDR plasmid from a clone is not unusual. However, what this study demonstrates is that the partial loss of a component of MDR plasmid may be accompanied by other changes in the genome, which increases virulence of the strain. Although the consequences of these changes in human was not apparent from this study it may be speculated that due to its increased virulence in the *Galleria* model, gastrointestinal or other infection with this strain could result in a different outcome. In such cases any subsequent treatment may be affected as this strain is resistant to 3^rd^ generation cephalosporin. We are currently assessing the effect of this strain, in comparison to the wild type ARD1258, in a chick model to determine any differences in colonisation which could subsequently influence its dissemination in humans through the food chain.
